# Temporal Progression of Recognition Memory Impairment, Astrogliosis, and Cholinergic Dysfunction in the Streptozotocin Rat Model of Sporadic Alzheimer’s Disease

**DOI:** 10.3390/ijms262210944

**Published:** 2025-11-12

**Authors:** Sofía Niño-Rivero, Rossana Cabral, Jazmín Fleitas, Lucía Alcalde-Ahlig, Eduardo M. Castaño, Laura Morelli, Ronald McGregor, Pablo Galeano, Patricia Lagos

**Affiliations:** 1 Unidad Académica de Fisiología, Facultad de Medicina, Universidad de la República, Gral. Flores 2125, Montevideo 11800, Uruguay; sofia.nino@pedeciba.edu.uy (S.N.-R.); rossanacabral1406@gmail.com (R.C.); jazzyfleitas@gmail.com (J.F.); lualcaldeahlig@gmail.com (L.A.-A.); 2 Laboratory of Brain Aging and Neurodegeneration, Fundación Instituto Leloir (IIBBA-CONICET), Av. Patricias Argentinas 435, Ciudad Autónoma de Buenos Aires C1405BWE, Argentina; ecastano@leloir.org.ar (E.M.C.); lmorelli@leloir.org.ar (L.M.); 3 Neuropsychiatric Institute and Brain Research Institute, University of California, Los Angeles, CA 90095, USA; 4 Neurobiology Research, Veterans Administration Greater Los Angeles Healthcare System, 16111 Plummer Street, North Hills, CA 91343, USA

**Keywords:** Alzheimer’s disease, streptozotocin, recognition memory, astrogliosis, acetylcholine, hippocampus, parietal cortex

## Abstract

The streptozotocin (STZ) experimental model of sporadic Alzheimer’s disease (SAD), the most prevalent form of this type of dementia, has become a valuable tool to study the behavioral and morphological changes that occur during the gradual development of this pathology. We used the STZ experimental model in combination with the novel object recognition test (NORT) and immunohistochemical techniques to evaluate the recognition memory decline and morphological alterations in memory-related structures (hippocampus and cortex). Our analysis included five different time points after intracerebroventricular (ICV) administration of 3 mg/kg of STZ or artificial cerebrospinal fluid (aCSF) as a control. The time points included three distinct stages: early (15 and 30 days), intermediate (60 days), and late (90 and 120 days). We found that recognition memory impairment started in the intermediate stage and persisted through the later stages. Morphologically, we detected significant astrogliosis starting in the early stages, whereas cholinergic changes began in the intermediate stage. No neuronal loss was observed at any of the time points analyzed. Our results further support the hypothesis that astrogliosis constitutes an initial pathological event that may compromise the hippocampal cholinergic system and can contribute to the onset of recognition memory deficits.

## 1. Introduction

Alzheimer’s disease (AD) is a progressive neurodegenerative disorder that leads to a form of dementia. It affects approximately 57 million individuals worldwide, according to the World Health Organization [1]. The estimated annual global cost of dementia is approximately US $1.3 trillion (based on 2019 data), with 60–70% of the cases being due to AD [2]. Low-to-middle-income countries are particularly affected by this financial burden. Reports for Latin America and the Caribbean have indicated that around 6.8 million people over 60 years of age were living with dementia in 2020, and that most of these cases correspond to AD. This number is expected to increase to 9.9 million by 2030 [3].

AD has been classified into two distinct forms based on the etiology of the disease. The familiar form (FAD), accounting for less than 5% of the cases, is characterized by early onset (EOAD) and has been linked to autosomal dominant mutations in genes encoding for Amyloid Precursor Protein (APP), Presenilin-1 (PSEN1), and Presenilin-2 (PSEN2) (reviewed in [4,5,6]). The sporadic form of AD (SAD) corresponds to the remaining 95% of the cases and has become the main focus of research given its prevalence [7]. SAD is characterized by late onset (LOAD) and is related to genetic factors, epigenetic alterations, and multiple environmental factors, including low educational levels, lifestyle factors, and exposure to pollutants [8,9,10].

Basic scientific research on AD has been mainly centered on the study of neurons. However, astrocytes, a subtype of glial cells that constitute the majority of cells in the central nervous system (CNS), have been increasingly recognized as major players in the development of AD in recent years [11]. Astrocytes, often characterized by the expression of glial fibrillary acidic protein (GFAP) [12], regulate synaptic activity and provide metabolic support and structural integrity to neurons [13]. Additionally, astrocytes are activated during inflammatory responses, increasing GFAP production and releasing neurotoxic factors [11,14,15]. Reactive astrocytes are thought to actively participate in Alzheimer-related neuroinflammation, not only through the secretion of inflammation-associated mediators but also via the production of reactive oxygen species and nitric oxide, ultimately leading to a redox imbalance [11,16].

Several experimental animal models have been developed to study SAD [17,18], including the bilateral intracerebroventricular (ICV) injection of streptozotocin (STZ) [19,20,21]. This model has become a valuable tool in understanding the progression of SAD, since it replicates several features of human SAD, including neuroinflammation, progressive memory impairment, and cholinergic dysfunction [22,23,24]. Behaviorally, STZ administration results in a progressive decline in learning and memory, including working and reference memory [19], non-declarative connecting memory [21,25], and spatial learning and recognition memory [26]. Morphologically, STZ affects both astrocytes and cholinergic neurons [20,27,28]. Earlier reports have documented upregulation of both GFAP transcript and protein expression, together with enhanced secretion of inflammatory cytokines IL-1β, IL-6, and TNF-α [20,29,30,31,32]. In addition, temporal variations in acetylcholinesterase (AChE) activity and choline acetyltransferase (ChAT) expression were observed in the hippocampus and cortex [20]. These findings are partly consistent with previous *postmortem* studies in humans diagnosed with mild cognitive impairment (MCI) and AD [33,34].

Previous reports using the STZ model have described morphological and neurochemical alterations at different temporal windows, ranging from early (14–30 days post-STZ) [26,35,36] to long-term stages (from 90 days and up to 9 months post-STZ) [20,21,37,38]. Early studies consistently reported marked astrocytic activation and increased expression of inflammatory mediators in cortical and hippocampal areas within the first weeks following STZ administration, indicating an early onset of neuroinflammatory processes [27,28,29]. At later stages, several groups have shown persistent gliosis, progressive cholinergic alterations, and synaptic or structural remodeling in hippocampal and cortical regions [21,30,37]. However, intermediate time points between one and three months post-STZ remain much less explored, limiting our understanding of how early glial activation evolves into later neuronal and cholinergic dysfunction. Moreover, although the Cornu Ammonis 1 (CA1) and Cornu Ammonis 3 (CA3) hippocampal subfields have been examined, the dentate gyrus (DG) has been less frequently characterized in this model. Therefore, the simultaneous assessment of CA1, CA3, and DG provides additional and complementary insight into the spatial heterogeneity of hippocampal responses, particularly at intermediate stages of the development of the STZ model in rats.

This present study was designed to provide a detailed temporal characterization of the development of recognition memory impairments and associated morphological alterations in brain regions associated with memory functions upon administration of STZ. We employed five distinct time points post-STZ administration that include early (15 and 30 days after STZ), intermediate (60 days after STZ), and late (90 and 120 days after STZ), and examined the parietal cortex and the hippocampal formation (CA1, CA3, and DG) in all experimental groups.

## 2. Results

### 2.1. Deficits in Recognition Memory Are Seen After 60 Days of STZ Administration

**Figure 1:**
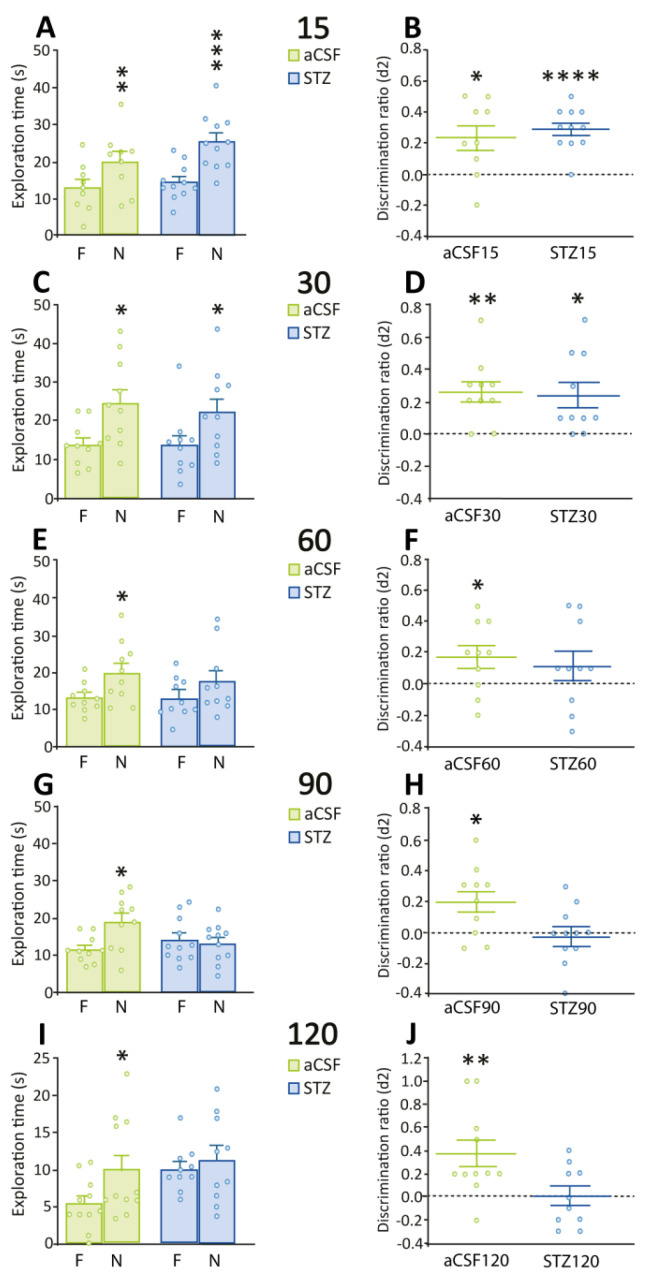
Performance in the retention sessions of NORT for STZ-treated and control rats. Animals were subjected to the NORT at (**A**,**B**) 15 days, (**C**,**D**) 30 days, (**E**,**F**) 60 days, (**G**,**H**) 90 days, and (**I**,**J**) 120 days after ICV administration of aCSF or STZ. (**A**,**C**,**E**,**G**,**I)** panels show the time that rats explored familiar (F) and novel (N) objects, * *p* ≤ 0.05; ** *p* ≤ 0.01; *** *p* ≤ 0.001 vs. time exploring F object. (**B**,**D**,**F**,**H**,**J**) panels show the discrimination ratios (d2) for all the experimental groups, * *p* ≤ 0.05; ** *p* ≤ 0.01; **** *p* ≤ 0.0001 vs. a hypothetical mean value of zero. Data are shown as the mean ± SEM of 9–11 rats per group with individual values superimposed.

Fifteen and thirty days after ICV administration of STZ or aCSF, treated rats explored the novel object for significantly more time than the familiar one in the retention session of the NORT (paired t-tests: aCSF 15: t = 3.46, *p* = 0.0086; STZ 15: t = 5.70, *p* = 0.0002; aCSF 30: t = 3.11, *p* = 0.0126; STZ 30: t = 2.62, *p* = 0.0277) (Figure 1A,C). d2 values were also significantly higher than zero (one-sample t-tests: aCSF 15: t = 2.92, *p* = 0.0193; STZ 15: t = 7.02, *p* < 0.0001; aCSF 30: t = 4.09, *p* = 0.0027; STZ 30: t = 3.09, *p* = 0.0130) (Figure 1B,D).

**Figure 2:**
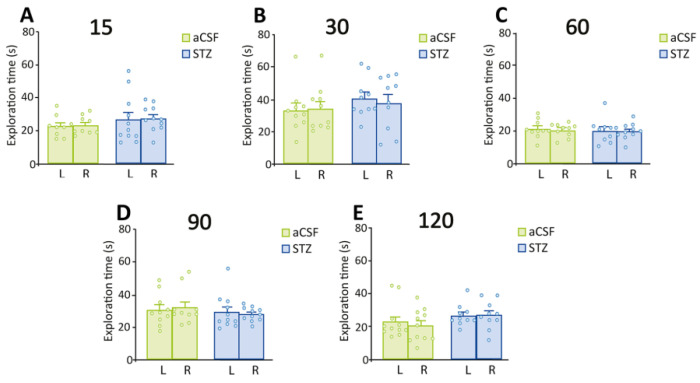
Performance in the training sessions of NORT for STZ-treated and control rats. Animals were subjected to NORT at (**A**) 15 days, (**B**) 30 days, (**C**) 60 days, (**D**) 90 days, and (**E**) 120 days after ICV administration of STZ or aCSF. No significant difference was observed in the time rats spent exploring the two identical objects, positioned on the left (L) and right (R) sides of the OF (paired t-tests. (**A**), aCSF15: t = 0.30; STZ15: t = 0.09; (**B**), aCSF30: t = 0.34; STZ30: t = 0.62; (**C**), aCSF60: t = 0.31; STZ60: t = 0.17; (**D**), aCSF90: t = 0.34; STZ90: t = 0.39; (**E**), aCSF120: t = 1.16; STZ120: t = 0.10. Data are shown as the mean ± SEM of 9–11 rats per group with individual values superimposed.

Similarly, 60, 90 and 120 days after ICV administration of aCSF, rats explored the novel object significantly more time than the familiar one (paired t-tests: aCSF 60: t = 2.54, *p* = 0.0318; aCSF 90: t = 3.24, *p* = 0.0101; aCSF 120: t = 3.08, *p* = 0.0117) (Figure 1E,G,I). On the contrary, STZ-treated rats spent similar amounts of time exploring the novel and familiar objects (Figure 1E,G,I). d2 values were significantly higher than zero in the aCSF groups (one-sample t-tests: aCSF 60: t = 2.37, *p* = 0.0415; aCSF 90: t = 2.79, *p* = 0.0208; aCSF 120: t = 3.22, *p* = 0.0092), but not in the STZ-treated groups (Figure 1F,H,J). During training sessions with two identical objects, rats from all experimental groups (aCSF: 15 to 120 days; STZ: 15 to 120 days) exhibited comparable exploration times for both objects (Figure 2).

### 2.2. Early Onset of Astrogliosis in Hippocampal Subfields CA1 and CA3

**Figure 3:**
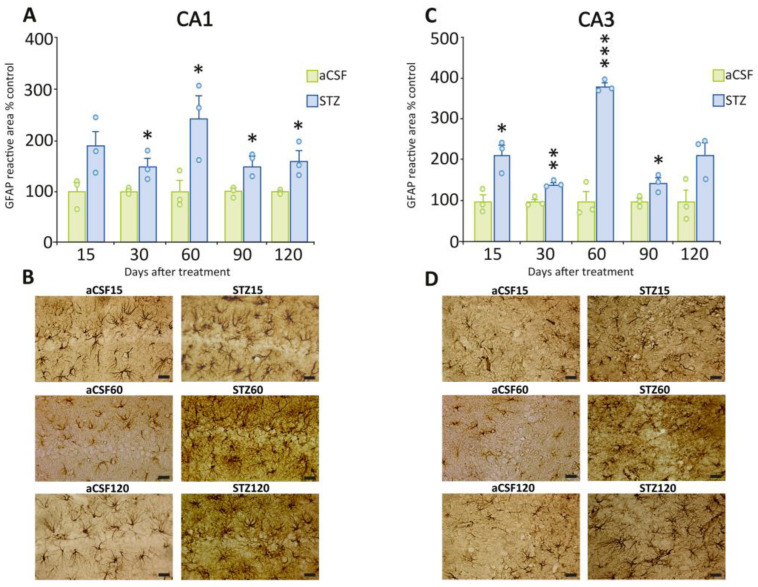
GFAP immunoreactivity in CA1 and CA3 regions from STZ-treated and control rats. (**A**) Quantification of the GFAP-immunoreactive area in the CA1 region at different time points after STZ or aCSF ICV administration. STZ-treated rats exhibited a significant increase in the GFAP-immunoreactive area at 30, 60, 90, and 120 days post-STZ compared to those treated with aCSF. (**B**) Representative photomicrographs of GFAP immunostaining in the CA1 region at 15, 60, and 120 days post-STZ or aCSF. (**C**) Quantification of the GFAP-immunoreactive area in the CA3 region at different time points after STZ or aCSF ICV administration. STZ-treated rats exhibited a significant increase in GFAP-immunoreactive area at 15, 30, 60, and 90 days compared to those treated with aCSF. (**D**) Representative photomicrographs of GFAP immunostaining in the CA3 region at 15, 60, and 120 days post-STZ or aCSF. Data are depicted as mean  ± S.E.M. Individual data points (n = 3 rats/group/time point) are also shown * *p* ≤ 0.05; ** *p* ≤ 0.01; *** *p* ≤ 0.001 vs. rats treated with aCSF. Scale bars = 20 µm.

We used GFAP, a widely used marker for astrocytes, that also serves as a reliable indicator of neuroinflammation due to its increased expression under inflammatory conditions (see Section 4) [14,15]. STZ treatment resulted in a significant increase in the GFAP immunoreactive area compared to aCSF in CA1 and CA3 regions of the hippocampus (Figure 3A, CA1, 30 days: t = 2.90, *p* = 0.0438; 60 days: t = 2.98, *p* = 0.0406; 90 days: t = 3.72, *p* = 0.0204; 120 days: t = 2.95, *p* = 0.0419; Figure 3C, CA3, 15 days: t = 4.21, *p* = 0.0135; 30 days: t = 6.17, *p* = 0.0035; 60 days: t = 11.25, *p* = 0.0004; 90 days: t = 3.19, *p* = 0.0331). The maximum increase in these two areas was seen 60 days post-STZ as compared to the aCSF groups (CA1 = 145%; CA3 = 283%). Figure 3B,D show representative images of CA1 and CA3 regions showing the increase in GFAP immunoreactive staining.

### 2.3. Delayed Astrogliosis in the Dentate Gyrus and Cerebral Cortex

**Figure 4:**
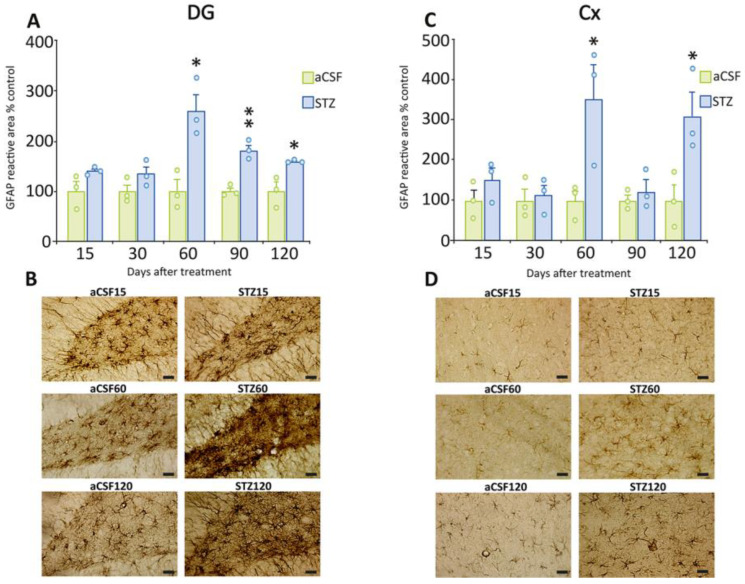
GFAP immunoreactivity in the DG of the hippocampal formation and cerebral cortex. (**A**) Quantification of the GFAP-immunoreactive area in the DG region at different time points after STZ or aCSF ICV administration. STZ-treated rats had a significant increase in GFAP-immunoreactive area at 60, 90, and 120 days compared to those treated with aCSF. (**B**) Representative photomicrographs of GFAP immunostaining in the DG at 15, 60, and 120 days post-STZ or aCSF. (**C**) Quantification of the GFAP-immunoreactive area in the Cx post-STZ or aCSF. STZ-treated rats exhibited a significant increase in GFAP-immunoreactive area at 60 and 120 days compared to those treated with aCSF. (**D**) Representative photomicrographs of GFAP immunostaining in the Cx region at 15, 60, and 120 days post-STZ or aCSF. Data are depicted as mean  ± S.E.M. Individual data points (n = 3 rats/group/time point) are also shown * *p* ≤ 0.05; ** *p* ≤ 0.01 vs. rats treated with aCSF. Scale bars = 20 µm.

In the DG and cerebral cortex, we also observed significant astrogliosis, but it began at later time points (from 60 days onwards) than in the CA1 and CA3 regions (Figure 4A, DG, 60 days: t = 4.07, *p* = 0.0152; 90 days: t = 6.90, *p* = 0.0023; 120 days: t = 3.53, *p* = 0.0241; Figure 4C, 60 days: t = 2.87, *p* = 0.0453; 120 days: t = 2.92, *p* = 0.0429). Interestingly, the maximum increase in these areas was also observed 60 days post-STZ treatment (DG = 162%; Cx = 254%). Figure 4B,D shows representative images of the DG and Cx regions, illustrating the increase in GFAP immunoreactivity.

### 2.4. The Cholinergic System in the Hippocampus Was Significantly Affected by STZ

**Figure 5:**
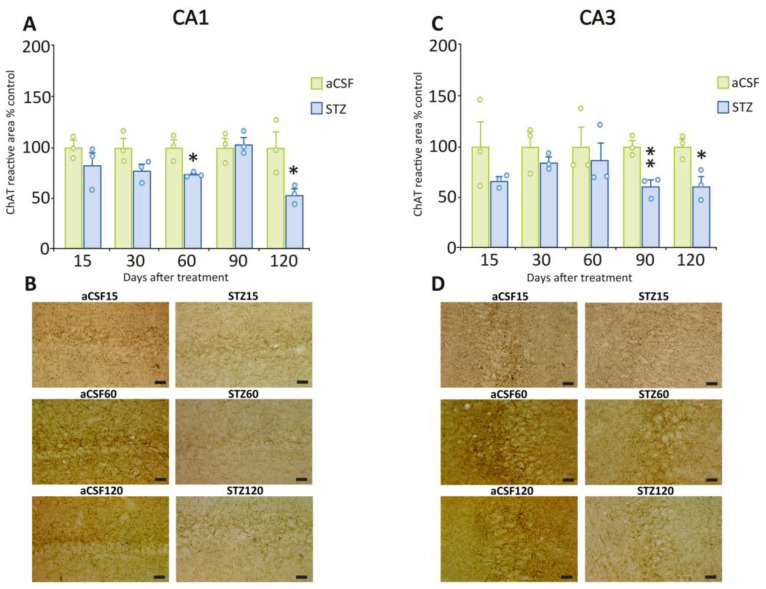
ChAT immunoreactivity in CA1 and CA3 regions from STZ-treated and control rats. (**A**) Quantification of the ChAT-immunoreactive area in the CA1 region at different time points post-STZ or aCSF. STZ-treated rats exhibited a significant decrease in ChAT-immunoreactive area at 60 and 120 days compared to those treated with aCSF. (**B**) Representative photomicrographs of ChAT immunostaining in the CA1 region at 15, 60, and 120 days post-STZ or aCSF. (**C**) Quantification of the ChAT-immunoreactive area in the CA3 region at different time points after aCSF or STZ. STZ-treated rats exhibited a significant decrease in ChAT-immunoreactive area at 90 and 120 days compared to those treated with aCSF. (**D**) Representative photomicrographs of ChAT immunostaining in the CA3 region at 15, 60, and 120 days post-STZ or aCSF. Data are depicted as mean ± S.E.M. Individual data points (n = 3 rats/group/time point) are also shown * *p* ≤ 0.05; ** *p* ≤ 0.01 vs. rats treated with aCSF. Scale bars = 20 µm.

**Figure 6:**
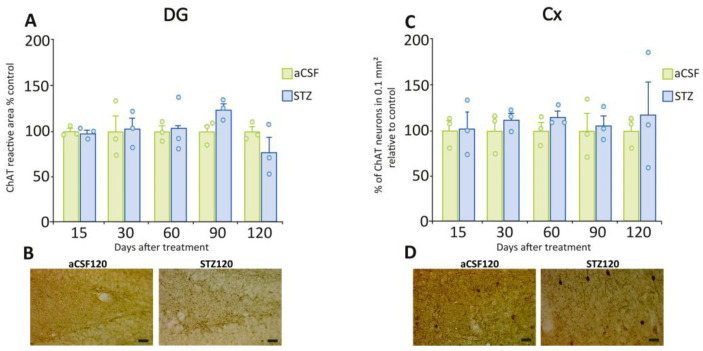
ChAT immunoreactivity in fibers of the DG of the hippocampal formation and in cholinergic neurons in the cerebral cortex. (**A**) Quantification of the ChAT-immunoreactive area in the DG at different time points after STZ or aCSF ICV administration. At no time point did the groups differ significantly. (**B**) Representative photomicrographs of ChAT immunostaining in the DG region at 120 days post-STZ or aCSF. (**C**) Number of cholinergic neurons in Cx (as % of ChAT neurons in 0.1 mm^2^ relative to control) at different time points post-STZ or aCSF. At no time point did the groups differ significantly. (**D**) Representative photomicrographs of ChAT immunostaining in the Cx at 120 days post-STZ or aCSF. Data are depicted as mean ± S.E.M. Individual data points (n = 3 rats/group/time point) are also shown. Scale bars = 20 µm.

We analyzed the presence of ChAT to visualize and quantify cholinergic neurons and fibers in the hippocampus. We observed a significant reduction in cholinergic fiber density (evaluated as density of ChAT immunoreactive area) post-STZ administration in hippocampal regions CA1 (Figure 5A, 60 days: t = 3.51, *p* = 0.0246; 120 days: t = 2.89, *p* = 0.0444) and CA3 (Figure 5C, 90 days: t = 4.87, *p* = 0.0082; 120 days: t = 3.71, *p* = 0.0206) compared to aCSF-treated rats. This decrease was comparable in both regions at 120 days post-STZ treatment (CA1 = 46% and CA3 = 39%). Figure 5B,D shows representative images of CA1 and CA3 hippocampal regions post-aCSF or post-STZ illustrating the observed changes in ChAT immunoreactivity. We did not detect differences in ChAT immunoreactive fiber density in DG (Figure 6A,B), or in the number of ChAT-positive neurons in the Cx (Figure 6C,D), post-STZ as compared to the aCSF groups.

### 2.5. STZ Administration Did Not Induce Neuronal Loss

**Figure 7:**
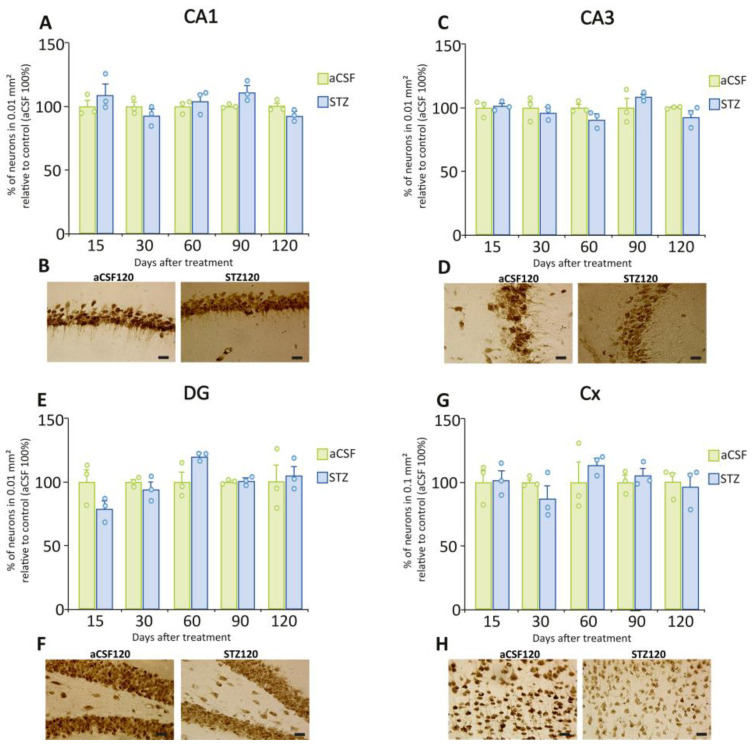
NeuN immunoreactivity in the hippocampal formation and cerebral cortex. (**A**) Number of NeuN-positive neurons in the CA1 region after STZ or aCSF ICV administration. At no time point did the groups differ significantly. (**B**) Representative photomicrographs of NeuN immunostaining in the CA1 region at 120 days post-STZ or aCSF. (**C**) Number of NeuN-positive neurons in the CA3 region after aCSF or STZ. At no time point did the groups differ significantly. (**D**) Representative photomicrographs of NeuN immunostaining in the CA3 region at 120 days post-STZ or aCSF. (**E**) Number of NeuN-positive neurons in the DG after STZ or aCSF ICV administration. At no time point did the groups differ significantly. (**F**) Representative photomicrographs of NeuN immunostaining in the DG at 120 days post-STZ or aCSF. (**G**) Quantification of the NeuN-positive neurons in the Cx at different time points post-STZ or aCSF. At no time point did the groups differ significantly. (**H**) Representative photomicrographs of NeuN immunostaining in the Cx at 120 days post-STZ or aCSF. In A, C and E the number of NeuN-positive neurons is expressed as % of ChAT neurons in 0.01 mm^2^ relative to control, while in G is expressed as % of ChAT neurons in 0.1 mm^2^ relative to control. Data are depicted as mean ± S.E.M. Individual data points (n = 3 rats/group/time point) are also shown. Scale bars = 20 µm.

To quantify neuronal cell number, we used NeuN, a reliable marker for mature neurons in the CNS of vertebrates and widely used for this purpose. We did not observe differences in the total number of NeuN-positive neurons in all areas analyzed (CA1, CA3, DG, and Cx) in STZ-treated compared to aCSF-treated animals (Figure 7).

## 3. Discussion

Animal models of SAD, including the ICV administration of STZ used in this study, have been developed to reproduce some of the key elements observed in humans with SAD. One of the advantages of using this interventional experimental SAD model in rats is that it allows the study of the gradual and slow progression of recognition memory-associated deficits, as well as the changes in morphological characteristics observed in different brain regions. The combination of these two experimental approaches allows the establishment of a link between behavior and morphology, providing a deeper understanding of the mechanisms underlying some of the key features of SAD. In this study, we performed a comprehensive time-course analysis of the STZ model in male rats that included two early time points (15 and 30 days post-STZ administration), one intermediate time point (60 days post-STZ administration), and two late time points (90 and 120 days post-STZ administration).

### 3.1. Behavioral Studies

Our behavioral experiments, using NORT, showed that the ICV administration of STZ (3 mg/kg) led to a progressive decline in recognition memory that was detectable at 60 days post-STZ and remained until 120 days post-STZ. It is important to note that all five cohorts of rats spent similar amounts of time exploring both identical objects during the training session (TS), discarding the possibility of object preference bias related to the location of the objects in the arena. Prior studies using the same test have shown similar findings, reporting behavioral deficits at 120 days post-STZ [39]. However, Afshar et al. [40] reported cognitive deficits as early as 30 days post-STZ, suggesting there can be some variability in the onset of the memory deficits in this experimental model. Memory deficits were also observed between 30 and 90 days post-STZ using the Morris water maze, a classical behavioral test used to evaluate spatial memory that depends on the dorsal hippocampus. Together, these results emphasize the capacity of the model to reproduce certain characteristics related to memory deficits observed in humans diagnosed with SAD [41,42].

Knezovic et al. [21] also evaluated the progressive cognitive and morphological changes resulting from different doses of ICV STZ at different time points. Using the passive avoidance test to evaluate associative and aversive memory, both of which are dependent on amygdala and hippocampal function [43,44], the authors defined an acute-response (up to 3 months post-STZ) and a chronic-progressive response (after 3 months post-STZ). This study further extends the behavioral and anatomical analysis by including 5 different time points, specifically incorporating an intermediate time point at 60 days. Our results show the importance of including this intermediate time point for future research related to the progression of cognitive impairment involving recognition memory. In addition, they offer further support for the use of the STZ model as a means to study cognitive dysfunction, particularly related to recognition and spatial and associative memory.

### 3.2. Immunohistochemical Studies

Using immunohistochemical techniques, we observed that STZ administration induces astrogliosis, indicated by a significant increase in GFAP immunoreactivity that started at early time points in CA3 and CA1, whereas the DG and the parietal cortex showed similar changes, although those were detected from the intermediate time point onwards. Additionally, we observed a significant decrease in cholinergic fiber density in specific subregions of the hippocampus (CA1 and CA3), particularly at intermediate and late time points, without an effect on neuronal cell counts in the hippocampus and the cortex. The results obtained in the CA3 region are in line with prior work showing an increase in GFAP protein levels and transcripts at 15 days post-STZ in the whole hippocampus [45], leading to the speculation that this increase could be related to synaptic loss. The presence of hippocampal astrogliosis as early as 15 days post-STZ in CA3 aligns with several studies identifying astrocyte reactivity as one of the initial pathological responses to the ICV administration of STZ [27,28,29,46,47,48]. It is important to note that the CA3 region has been identified as a key player in mnemonic processes (for review, see [49]). The increase in GFAP+ signals observed at that early time point persisted at intermediate and late time points in CA1, CA3, and DG. However, in the parietal cortex, astrogliosis appeared at the intermediate time point compared to control groups, and was also observed at 120 days post-STZ. These results suggest a region-specific progression of morphological modifications that could potentially be related to different sensitivities of distinct types of astrocytes located in each hippocampal subregion or the cortex to the treatment. This speculation is supported by a study that employed different approaches to label astrocytes in the adult CNS of mice and determined the existence of regional and sub-regional heterogeneity in astroglial morphology, density, and proliferation, indicating that they represent a highly diverse type of glial cells [50]. Thus, we hypothesize there might be functional differences in astrocytes’ sensitivity to ICV STZ depending on their location, morphology, and density.

Several non-mutually exclusive factors may underlie the earlier astrocytic reactivity observed in CA3 compared with the DG in our model. Astrocytes display substantial molecular and functional heterogeneity across brain regions, and subfield-specific signaling or transcriptional programs could contribute to distinct reactive dynamics [51]. Empirical evidence from transgenic models also shows regional differences in glial responses and neurodegenerative patterns among CA1, CA3, and DG, with CA3 often exhibiting stronger or earlier glial activation [52]. Given the high excitatory drive, dense recurrent connectivity, and greater metabolic demand of CA3 circuits, disturbances in astrocytic homeostatic functions, such as glutamate or K^+^ buffering, may produce earlier structural and functional changes in this subfield. In addition, context-dependent astrocyte-microglia crosstalk and complement-mediated signaling have been implicated in regionally biased glial responses and selective synapse vulnerability [53]. Together, these observations support a plausible model in which intrinsic astrocyte heterogeneity, higher excitatory/metabolic load in CA3, and differential glia-immune interactions contribute to earlier astrocyte reactivity in CA3, whereas the DG, characterized by distinct cellular architecture, ongoing neurogenesis, and different trophic conditions, exhibits similar changes at later stages.

Astrocyte activation sustained over time likely creates a chronic cytotoxic environment that promotes synaptic disturbance and memory dysfunction [11,14,28]. Notably, STZ induces the release of IL-1β, IL-6, and TNF-α in mice, all pro-inflammatory mediators that contribute to neuronal dysfunction [30]. Recent evidence showed a reduction in astrogliosis and improved cognition with melatonin treatment, supporting the use of the STZ model in the screening of anti-neuroinflammatory drugs [54].

Reactive astrocytes can disrupt synaptic environments when they lose their functions as trophic support to neurons and oligodendrocytes and as active partners in tripartite neurotransmission [15,55]. The persistent presence of reactive astrocytes could impair glutamate uptake and GABA recycling, potentially leading to progressive excitotoxicity that may compromise cholinergic circuit integrity and synaptic functioning [56,57,58,59].

In our work, we observed that the astrogliosis in CA1 and CA3 precedes the appearance of recognition memory deficits and the decreased cholinergic fiber density, highlighting the relevance of astrocytes in the regions related to memory processes. This suggests that astrocytes could be one of the first types of cells to be affected by STZ administration. One plausible explanation for the temporal sequence we observe (early astrocyte reactivity in CA1/CA3 followed by later loss of ChAT+ fibers) is that reactive astrocytes progressively shift from a supportive to a neurotoxic state, activating several complementary molecular cascades that impair, over time, the cholinergic terminals that were in the parenchyma adjacent to such reactive astrocytes. First, it was observed that reactive astrocytes lose homeostatic functions, including glutamate and potassium buffering, in particular through the downregulation of glutamate transporters and Kir4.1-mediated K^+^ buffering, leading to increased extracellular glutamate and excitotoxic stress on neighboring synapses [60]. Second, they release pro-inflammatory mediators (e.g., IL-1β, TNF-α) and complement components such as C3, which can tag synapses and sustain microglial phagocytosis, promoting delayed pruning of axonal terminals [61,62]. Third, subsets of A1-like astrocytes produce reactive oxygen and nitrogen species and engage pathways associated with lipid peroxidation and ferroptosis, processes shown to occur in astrocytes and to promote local oxidative damage and synaptic vulnerability [63,64,65,66]. Such oxidative and ferroptotic stressors can impair axonal terminals and synapses, including cholinergic presynaptic sites, and may precede overt neuronal loss, although direct evidence of this sequence in hippocampal cholinergic fibers remains limited. In parallel, reactive astrocytes undergo metabolic reprogramming and reduce the release of neurotrophic and metabolic factors (e.g., BDNF, NGF, and lactate), thereby compromising trophic support to cholinergic terminals that were diminished in density over time, and that further could contribute to synaptic vulnerability [67,68]. Collectively, these mechanisms offer a coherent molecular framework by which early astrocytic activation may progressively compromise cholinergic integrity, consistent with the temporal pattern observed in our study.

The disturbances in the cholinergic system are one of the long-standing mechanisms related to AD [69]. This idea is based on the observation that cholinesterase inhibitors have positive effects in aged humans as well as in patients with dementia. We observed a significant reduction in cholinergic fiber density in CA1 and CA3 in the intermediate and late time points post-STZ administration. Both hippocampal subregions are involved in memory consolidation and retrieval processes, and thus, the decrease in cholinergic innervation could be one of the factors underlying the recognition memory deficits that became significant at the intermediate time point of 60 days after STZ. In line with this, Davies and Maloney [70] reported a marked reduction of ChAT and AChE activity in the hippocampus of AD patients, supporting the view that the cholinergic system is selectively affected in this condition.

We did not find a significant modification in the number of ChAT-positive neurons in the parietal cortex across our experimental groups as compared to controls. In line with our results, the activity of AChE and butyrylcholinesterase (BChE) remained unchanged in the prefrontal cortex of rats evaluated at 30 and 120 days post-STZ [38]. In addition, AChE activity showed a significant decrease in the hippocampus at both time points; meanwhile, the diminution of BChE activity was found only at the late time point. This suggests the possibility that the cholinergic system in the STZ model could be differentially affected depending on both the structure of the CNS and the presence of cholinergic neurons or fibers. Taken together, these results support the importance of the modifications to the cholinergic system in the hippocampus related to memory processes in this model of SAD in male rats. We speculate that cholinergic fibers are more sensitive than neurons to the effects of reactive astrocytes at the different subregions of the hippocampus. Moreover, the occurrence of astrogliosis preceded the decrease in the density of cholinergic fibers in CA1 and CA3.

Importantly, we observed a decrease in the density of cholinergic fibers in CA1 paired with the recognition memory impairment at the intermediate time point. This temporal correspondence aligns with the role of cholinergic signaling in memory processes [71,72]. Prior work using Western blot to quantify protein levels has shown a decrease in hippocampal ChAT levels at 7 days post-STZ, followed by a compensatory increase at 30 days that remained stable for up to nine months [20]. In addition, Biasibetti et al. [27] observed a significant decrease in hippocampal ChAT expression at 15, 30, and 60 days post-STZ. In this study, the use of immunohistochemistry to detect ChAT enabled the analysis of specific regions and subregions of larger structures (e.g., hippocampal CA1, CA3, and DG), enabling a granularity that cannot be obtained using Western blot procedures on whole-hippocampus homogenates.

Despite these astrocytic and cholinergic alterations, we found no evidence of widespread neuronal loss in hippocampal subregions or the parietal cortex. Nevertheless, our findings are consistent with other studies that reported limited neurodegeneration by fluoro-Jade or Nissl stain at different time points of the STZ model [21,47]. This contrasts with studies by Chen et al. [73], who observed significant loss of NeuN-positive neurons in CA1 and CA3 at ~45 days post-STZ, and by Zappa Villar et al. [48], who reported CA1 hippocampal atrophy at 25 days post-STZ. These differences could be since these studies used different doses of STZ, as well as different rat or mice strains.

While previous studies have explored aspects of the pathology between 7 days and 9 months post-STZ [20,21], our work provides a higher temporal profile, further supporting an STZ model that has been previously suggested [47], in which an initial astrogliosis could affect the cholinergic system at the hippocampus, and induce the appearance of cognitive deficits.

### 3.3. Limitations of the Study

Although the present study provides a detailed temporal profile of recognition memory alterations in the STZ model, we acknowledge that only one behavioral test was employed, the NORT. This choice was based on the need to evaluate specific post-treatment time-points (15, 30, 60, 90, and 120 post-STZ treatment). Tests requiring prolonged training would have blurred the precision of these temporal assessments. Nevertheless, other short-duration behavioral paradigms, such as the Object Location Test, the Y-Maze spontaneous alternation task, among others, could be incorporated in future studies to complement the present findings and further characterize cognitive progression in this model.

Another limitation of this work is that only male rats were evaluated. Previous reports employing female subjects have shown differences compared to males, and the presence of ovaries significantly affects the response to STZ, emphasizing the complexity of sex-related factors in this model [27,74]. Considering both sexes would have required doubling the total number of animals, which was beyond our practical capacity. Alternatively, keeping the same total number of animals per group but splitting each group equally by sex would have reduced the statistical power and made it difficult to clearly distinguish sex-specific effects. Moreover, hormonal fluctuations in females can introduce additional variability that may mask treatment-related differences. Nonetheless, our ongoing and unpublished work includes female cohorts evaluated at different time points post-STZ using behavioral tests and biochemical analyses to assess neuroinflammatory mediators like cytokines, which will allow us to address these sex-related aspects in future reports.

## 4. Materials and Methods

### 4.1. Subjects

All experimental protocols received authorization from both the Institutional Commission for Animal Care (approval code 070153-000011-17, 8 September 2017) and the Bioethics Committee of the Facultad de Medicina (Universidad de la República, UdeLAR, Montevideo, Uruguay). The study was conducted in accordance with the Uruguayan legislation regulating experimentation with animals (N° 18.611) and adhered to the recommendations described in the “Guide for the Care and Use of Laboratory Animals” [75].

The study was carried out using adult Wistar male rats weighing between 250 and 300 g, beginning once they reached approximately two months of age. Animals were housed under controlled environmental conditions, with the temperature stabilized at 22 ± 2 °C, and exposed to a 12-h light/dark schedule (illumination from 6:00 AM to 6:00 PM). Food and water were provided ad libitum throughout the experimental period.

### 4.2. Drugs

Streptozotocin (STZ; Sigma-Aldrich, S0130, St. Louis, MO, USA) was dissolved in aCSF (artificial cerebrospinal fluid), which was prepared according to Nitsch and Hoyer [76]. aCSF (vehicle or control) and STZ were administered by intracerebroventricular injection (ICV, see below).

### 4.3. Surgical Procedures

**Figure 8:**
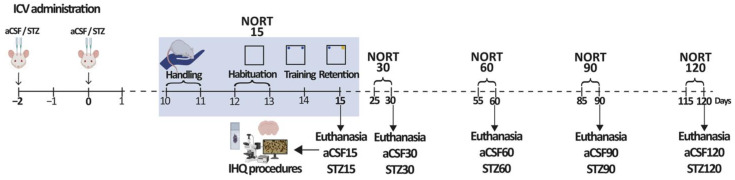
Schematic timeline of experimental design. Five cohorts of male rats were used: cohort 1 (aCSF15, STZ15), cohort 2 (aCSF30, STZ30), cohort 3 (aCSF60, STZ60), cohort 4 (aCSF90, STZ90), and cohort 5 (aCSF120, STZ120). Rats received two bilateral ICV injections of aCSF or STZ, the first on day −2 and the second on day 0, for a total dose of 3 mg/kg of STZ. NORT was conducted after 15, 30, 60, 90, or 120 days. An example of the different sessions of NORT evaluated in the 15-day groups is shown inside a light blue rectangle: handling, habituation, training, and retention. After the retention session, animals were anesthetized, and their brains were fixed and processed for immunohistochemical experiments.

Animals received bilateral ICV injections of aCSF or STZ (3 mg/kg, total dose). Procedures were done twice with a 48 h period in between injections. (day −2 and day 0; see Figure 8, experimental design). We employed ketamine (90 mg/kg, i.p.) and xylazine (5 mg/kg, i.p.) to anesthetize the animals. Following verification that reflexes were absent, animals were positioned in a stereotaxic apparatus and the skull surface exposed. During the first surgery (day −2), two small craniotomies were drilled at stereotaxic coordinates targeting the lateral ventricles (AP: −0.9 mm; L: ±1.6 mm relative to bregma). Infusions of either STZ or aCSF were delivered with a Hamilton microsyringe inserted to a depth of −3.4 mm from the cortical surface. The injection rate was fixed at 1 µL/min, with a total of 8–10 µL administered per ventricle. We calculated the dose of STZ diluted in aCSF to obtain a final volume correlated with the body weight of each animal (9 μL/300 g). After infusion, the syringe remained in place for 7 min to prevent reflux before being carefully withdrawn. Correct ventricular placement was verified by the presence of freely flowing cerebrospinal fluid upon needle withdrawal. The incision was then closed with sutures and, once 45–60 min had elapsed, the animals were placed back in their home cage. On day 0, the second ICV administration was performed using the same procedures. At the end of the surgery, sterile bone wax was applied to cover both craniotomies, and the skin was again sutured. Post-operative care included monitoring and the administration of amoxicillin (0.05 mg/kg, s.c.) and ketoprofen (3 mg/kg, s.c.) on days −2, −1, 0, 1, and 2. After recovery, the rats were housed under standard conditions until the experimental endpoints, scheduled at 15, 30, 60, 90, and 120 days after the second injection of either aCSF or STZ.

### 4.4. Experimental Design

Five cohorts of animals were used, each consisting of a control (aCSF) and a treated (STZ) group. Behavioral and morphological analyses were performed at 15, 30, 60, 90, and 120 days following administration of aCSF or STZ. At the end of the behavioral procedures, rats were euthanized, their brains were dissected, and the tissue was processed for subsequent immunohistochemical studies. A schematic representation of the experimental design is shown in Figure 8.

### 4.5. Novel Object Recognition Test

All behavioral experimental sessions were carried out from 10 AM to 2 PM. In the present report, we adapted a version of NORT previously used by Galeano et al. [77], using a black open-field (OF) arena (80 × 80 × 40 cm) located in a behavioral room with the light intensity set between 9 and 15 lux. Two different object types were used during the task. One type consisted of two identical glass flasks of cylindrical shape, each 18 cm in height and decorated with white horizontal stripes. The other type consisted of two identical Erlenmeyer flasks, each 17 cm in height, featuring black vertical stripes. The objects were selected to prevent animals from pushing them or climbing on top of them.

### 4.6. Procedures

NORT was performed over six consecutive days (see Figure 8), and all sessions were videotaped with a Logitech webcam (model Pro Stream C922 Pro Full HD 30FPS, Lausanne, Switzerland). Two handling sessions of 3 min per animal were conducted on days 1 and 2, followed by two habituation sessions on days 3 and 4. During habituation sessions, each animal was positioned at the center of the OF and permitted to explore the arena freely for a duration of 10 min. On day 5, one training session (TS) was conducted during which two identical objects (cylindrical glass or Erlenmeyer flasks) were placed symmetrically in two of the corners that were on the same wall of the box. Animals were then placed in the OF, facing the wall opposite the objects, and allowed free exploration for 5 min. Twenty-four hours after the TS (on day 6), a retention session (RS) was carried out, where two objects were placed in the OF in the same location as the objects during the TS: one object was identical to the ones presented in the TS (the familiar), whereas the second object was a different one (the novel). For example, if the rat was presented with two cylindrical glass flasks in the TS, then in the RS, it was presented with one cylindrical glass flask and one Erlenmeyer flask. The objects presented during the TS were referred to as the familiar objects (FO), whereas the one presented at the RS was considered the novel object (NO). In the RS, each animal was positioned in the OF, oriented toward the wall opposite the objects, and allowed to freely explore both objects for 3 min. Rats with unimpaired cognition tend to spend more time exploring the NO than the FO due to their natural preference for novelty [78].

An experienced researcher, blind to the treatment condition, quantified the time spent exploring both objects using the videos obtained from the TS and the RS. A positive exploratory behavior was defined as the animal directing its snout toward the object at a proximity of 2 cm or less. Total exploration time for each object was recorded during both the TS and RS. Based on these measurements, the discrimination ratio (d2) for the RS was determined using the following formula: d2 = (tNO − tFO)/(tNO + tFO), where tNO represents the duration spent exploring the NO, and tFO represents the duration spent with the FO. To prevent olfactory cues from influencing subsequent animals, the OF and objects were thoroughly cleaned using ethanol (70%) and allowed to dry after each session.

### 4.7. Brain Fixation and Histological Procedures

After NORT behavioral sessions were finished, urethane (1.2 g/kg) was administered to anesthetize the rats before transcardial perfusion. Initially, approximately 0.3 L of heparinized PBS (0.1 M, pH 7.4) was perfused, followed by approximately 0.7 L of paraformaldehyde (PFA) at 4% prepared in PBS (0.1 M, pH 7.4). Brains were then carefully extracted and post-fixed at 4 °C overnight (ON) in 4% PFA. The tissue was then transferred to PB containing 30% sucrose in order to cryoprotect the brains. Forty-eight hours later, brains were rapidly frozen on dry ice and sectioned coronally at 30 μm using a cryostat (Leica CM 1900, Nussloch, Germany). Based on the Paxinos and Watson atlas [79], we collected brain sections from −2.8 to −4.16 mm from bregma. The sections were placed in a sequential way in a 12-well tray with a cryoprotectant solution and kept at −20 °C until used for immunohistochemical (IHC) analysis.

### 4.8. Immunohistochemical Procedures

Sections containing the dorsal hippocampus and parietal cortex were processed for the visualization of astrocytes, cholinergic neurons and fibers, and neuronal cell bodies using IHC detection of specific markers: GFAP (astrocytes), ChAT (cholinergic neurons and fibers), and the neuronal marker hexaribonucleotide binding protein-3 (NeuN) to identify neurons. Antibody specificity was confirmed by omission of the primary antibody.

All IHC procedures were performed at gentle agitation and using a series of incubations on free-floating sections, as detailed in our previous publications [80,81]. GFAP detection was performed using mouse anti-GFAP antibody (MS 1407P0, Thermo Scientific, Rockford, IL, USA, 1:1000). Detection of ChAT was performed with goat anti-ChAT antibody (AB144P, Millipore Sigma, St. Louis, MO, USA, 1:500). NeuN was identified using mouse anti-NeuN antibody (MAB377, Millipore Sigma, St. Louis, MO, USA, 1:1000).

Tissue sections were initially incubated for 30 min in 1% hydrogen peroxide (H_2_O_2_). This was followed by an ON incubation at room temperature (RT) with the primary antibody, prepared in PBS-T (PBS containing 0.3% Triton X-100). Subsequently, sections were exposed for 90 min at RT to the appropriate biotinylated secondary antibody (1:600) diluted in PBS-T with 1.5% normal donkey serum (Jackson ImmunoResearch, West Grove, PA, USA). The sections were then treated for 60 min at RT with the standard avidin-biotin complex (ABC, Vector Laboratories, Burlingame, CA, USA; 1:300) in PBS. Finally, the immunoreaction was visualized by immersing the sections in 0.02% DAB (diaminobenzidine tetrahydrochloride, SK-4100, Vector Laboratoriess, CA, USA) and 0.03% H_2_O_2_ in PBS for 10 min. IHC development using the DAB method was performed following a standardized protocol to minimize variability, as described in McGregor et al. [80]. All procedures were carried out on coded sections to ensure blinding of the experimenter.

### 4.9. Quantitative Analyses of IHC Assays

Quantification of GFAP, ChAT, and NeuN immunoreactivity was performed in three sections per animal. In each section, we analyzed the parietal cortex (Cx), the CA1, CA3 regions, and the dentate gyrus (DG) of the hippocampal formation. An Axiolab E microscope (Carl Zeiss, Oberkochen, Germany) equipped with a MU853B camera (Amscope, Hong Kong, China) was used to acquire all of the images. Quantitative analyses were performed by a histologist unaware of the treatment conditions, with the same individual evaluating both control (aCSF) and STZ sections.

**Figure 9:**
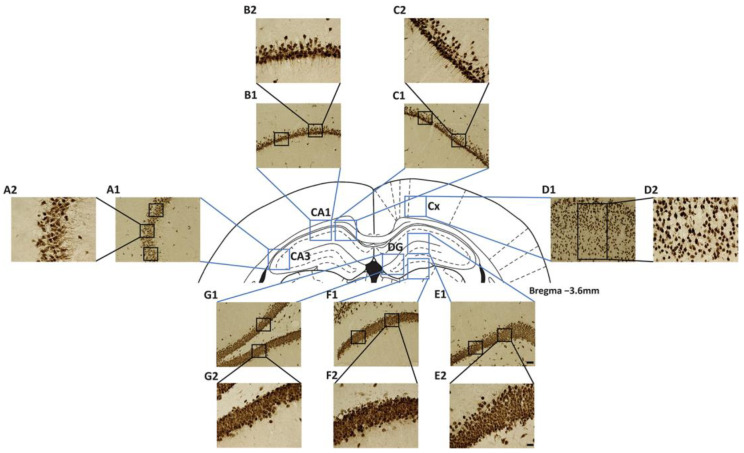
Diagram description of the localization of the images and regions of interest (ROI) obtained from selected regions from the four structures analyzed by immunohistochemistry: CA1, CA3, and DG of the hippocampal formation and parietal cortex (Cx). At the center of the figure is shown a diagram of the dorsal part of a representative coronal section obtained from −3.6 mm from bregma ([79]). Each light blue square points out the representative selected region or regions of each of the analyzed structures where 20× images were obtained. One unique 20× image was obtained for the quantification of positive immunoreactivity in CA3 (**A1**), where we selected three ROIs (black squares) to quantify the different markers. A high magnification image is shown from one of such ROIs (**A2**). In CA1, two 20× images were obtained (**B1,C1**), and in each image, two ROIs were selected to do quantification. A high magnification image is shown from one of such ROIs (**B2**,**C2**). For the Cx, one huge rectangular ROI was used for the quantification (**D1**); a higher magnification is shown (**D2**). Finally, in the case of the DG, three 20× images were obtained (**E1**,**F1**,**G1**), and in each one, two ROIs were used to obtain the quantification. A high magnification image is shown from one of such ROIs (**E2**,**F2**,**G2**). The selection of the area occupied by the ROIs in each 20× image and the quantification of GFAP, ChAT, and NeuN immunoreactivity were done with ImageJ software. Representative images of NeuN immunoreactive sections were used for this figure. The localization of the 20× images (light blue squares) in the coronal section and the ROIs (black squares or rectangles) inside each image is shown in a representative manner. Scale bar = 50 µm for 20× images (blue light squares), like E1 shows, and scale bar = 20 µm for the 40× images of the selected ROIs obtained from the 20× image, like E2 shows.

All measurements were obtained from 20× images by selecting multiple rectangular regions of interest (ROIs) within the selected structures (Figure 9), and the quantification was done using ImageJ software v2.14.0/1.54f (National Institutes of Health, Bethesda, MD, USA). The number of images obtained in each region was based on the size of the structure: for CA1, we selected two 20× images, for CA3 and Cx we included one 20× image, and for DG, we included three 20× images. In each image, the quantification of the markers was performed in a different number of ROIs: two ROIs were used for CA1 and DG regions; three ROIs for CA3, and one ROI for Cx.

### 4.10. Statistical Analysis

GraphPad Prism software (version 8.0.2; GraphPad Software, San Diego, CA, USA) was used to perform all statistical analyses. Paired t-tests were carried out to compare the time spent by rats exploring objects in NORT. Discrimination index (d2) was compared to a hypothetical mean value of zero using one-sample t-tests. Unpaired t-tests were used to analyze all the data related to IHC assays (% of positive-immunoreactive areas or number of ChAT or NeuN-positive neurons expressed as % of ChAT or NeuN neurons in the correspondent area relative to control). A *p*-value equal to or less than 0.05 (5%) was considered to be significant. Morphological values were expressed as a percentage of the mean value of the aCSF group (set to 100%). Data are depicted as mean ± S.E.M. Individual data points are also shown.

## 5. Conclusions

Based on our results, we propose the STZ model of SAD as one of hippocampal and cortical astrogliosis with cholinergic degeneration in brain regions involved in memory processes. Our study reinforces the utility of this model for investigating, in a more detailed manner, morphological alterations associated with a slow recognition memory decline across time, from early time points of 15 and 30 days, to an intermediate one of 60 days, to as late as 90 and 120 days post-STZ administration. This approach emphasizes the relevance of the inclusion of an intermediate time point in order to study astrogliosis and cholinergic dysfunction rather than classical neurodegeneration. Further investigation into the mechanisms by which STZ triggers sustained glial activation, cholinergic deficits, and their related cognitive consequences would be of significant interest.

## Data Availability

The raw data supporting the conclusions of this article will be made available by the authors on request.

## Abbreviations

The following abbreviations are used in this manuscript:

AD	Alzheimer’s Disease
SAD	Sporadic Alzheimer’s Disease
FAD	Familial Alzheimer’s Disease
EOAD	Early-Onset Alzheimer’s Disease
LOAD	Late-Onset Alzheimer’s Disease
Aβ	Amyloid-Beta
APP	Amyloid Precursor Protein
PSEN1/PSEN2	Presenilin 1/Presenilin 2
APOE	Apolipoprotein E
STZ	Streptozotocin
ICV	Intracerebroventricular
aCSF	Artificial Cerebrospinal Fluid
CA1/CA3	Cornu Ammonis 1/3
DG	Dentate Gyrus
Cx	Cortex
GFAP	Glial Fibrillary Acidic Protein
ChAT	Choline Acetyltransferase
NeuN	Hexaribonucleotide Binding Protein-3
NOR/NORT	Novel Object Recognition Test
PBS	Phosphate Buffered Saline
PFA	Paraformaldehyde
ABC	Avidin-Biotin Complex
DAB	Diaminobenzidine
ROI	Region of Interest
RT	Room temperature
SEM	Standard Error of the Mean
i.p.	Intraperitoneal
ON	Overnight
